# Gold nanoparticle decoration potentiate the antibacterial enhancement of TiO_2_ nanotubes *via* sonodynamic therapy against peri-implant infections

**DOI:** 10.3389/fbioe.2022.1074083

**Published:** 2022-11-17

**Authors:** Yue Sun, Wenzhou Xu, Cong Jiang, Tianyu Zhou, Qiqi Wang, Lan A

**Affiliations:** ^1^ Department of Oral Implantology, School and Hospital of Stomatology, Jilin University, Changchun, China; ^2^ Jilin Provincial Key Laboratory of Sciences and Technology for Stomatology Nanoengineering, Changchun, China; ^3^ Department of Periodontology, School and Hospital of Stomatology, Jilin University, Changchun, China

**Keywords:** sonodynamic therapy, titania nanotube, antibacterial, dental implant, ROS

## Abstract

Inflammatory damage from bacterial biofilms usually causes the failure of tooth implantation. A promising solution for this challenge is to use an implant surface with a long-term, in-depth and efficient antibacterial feature. In this study, we developed an ultrasound-enhanced antibacterial implant surface based on Au nanoparticle modified TiO_2_ nanotubes (AuNPs-TNTs). As an artificial tooth surface, films based on AuNPs-TNTs showed excellent biocompatibility. Importantly, compared to bare titania surface, a larger amount of reactive oxygen radicals was generated on AuNPs-TNTs under an ultrasound treatment. For a proof-of-concept application, *Porphyromonas gingivalis (P. gingivalis)* was used as the model bacteria; the as-proposed AuNPs-TNTs exhibited significantly enhanced antibacterial activity under a simple ultrasound treatment. This antibacterial film offers a new way to design the surface of an artificial implant coating for resolving the bacterial infection induced failure of dental implants.

## 1 Introduction

Safe, effective, and convenient therapeutic treatments are goals for nanomedicine development. For oral implantology, a titanium (Ti) dental implant is the most commonly used teeth substitution in the dental implant surgery owing to excellent biocompatibility. Unfortunately, it has still some shortcomings. The weak antibacterial activity of the titanium implant leads to patients suffering from complications and severely affects the success rate of implant treatment. Peri-implantitis, an inflammatory disease caused by bacterial biofilm, is the most common postoperative complication that leads to the failure of dental implant (Li et al., 2019). A conventional implant surface does not exhibit antibacterial activity, in contrast it accumulates bacteria and in turn facilitates the attachment loss and a rapid progress in peri-implantitis (Vargas-Reus et al., 2012). In spite of strict sterilizing procedures, bacterial contamination is difficult to avoid in an oral environment. As soon as the microbes arrive at the surface of implant, the extracellular viscous polysaccharide secreted by the aggregates of bacteria forms a biological film, which cause a high resistance to antibacterial agents and host defense. Ultimately, it leads to obstinate infections (Chung and Khanum, 2017; Skariyachan et al., 2018). To overcome the infection, endowing the surface of implants with a long-term and efficient antibacterial capability would be an effective measure, which can inhibit the bacterial colonization and succeeding formation of the biological film (Gao et al., 2014; Raphel et al., 2016; Zheng et al., 2020).

For decades, plenty of strategies have been used to enhance the antibacterial capability of titanium surfaces by modification with antibacterial agents such as antimicrobial peptides (Ma et al., 2012), metals ions (Zielińska-Jurek et al., 2015), polymers (Kubacka et al., 2007), and antibiotics (Kutuzova et al., 2021). In recent years, for minimal invasiveness and well-established clinical effect, reactive oxygen species (ROS)-associated antibacterial therapy has attracted much attention, such as photodynamic therapy (PDT) (Xu et al., 2019). PDT utilizes light of a specific wavelength to generate ROS *via* activating photosensitizers. ROS can cause cell membrane damage and subsequent death (Wang Z. et al., 2018) while stimulate the host immune system combat disease. Although PDT has been regarded as a hopeful substitutive strategy to traditional immunotherapy and chemotherapy, the low penetration (0.5–2.0 mm) of light radiation in biological tissue limits the application of PDT for deep tumors or infections in clinic (Wan et al., 2016). Therefore, sonodynamic therapy (SDT) has attracted much more attention, using therapeutic ultrasound instead of light to generate ROS, causing the apoptosis, necrosis, and autophagy of bacteria, together with mechanical death and necrosis caused by the implosion of cavitating bubbles (Zhu et al., 2021). On account of the low tissue attenuation and radiation-less nature, ultrasound can penetrate much thicker tissues without degradation of energy compared to light (Xu T. et al., 2020). Additionally, it passes through multi-layer of tissue without harming anything and stimulates the sensitizer only at its focus with a high spatial accuracy, whereas near-infrared light may excite all the sensitizers that appear during its propagation. Thus, all the advantages mentioned above confirm the safety of SDT when dealing with deep-seated diseases (Deepagan et al., 2016). Nevertheless, some challenges still limit the development and clinical application of SDT. For example, the quantum yield and biological safety should be the bran-new perspective to enhance the performance of the sonosensitizer, thus increasing the SDT efficiency.

Unlike organic sensitizers, an inorganic TiO_2_ nanostructure, a stable SDT agent of tolerance to oxidative degradation, could resist more ultrasound exposure time (Wei et al., 2013; Deepagan et al., 2016). Moreover, TiO_2_ nanostructures will not trigger the immune response as it is inert materials (El-Fray et al., 2009). When irradiated with energy from an external source, nano-TiO_2_ could generate ROS, containing superoxide radical (•O_2_^−^), hydroxyl radical (•OH), and singlet oxygen (^1^O_2_) (Wang et al., 2011; Ma et al., 2012; Wang et al., 2013). Nevertheless, there is a problem: The performance of pure TiO_2_ as a sensitizer is limited by the low yield of ROS, causing a fast electron-hole recombination (Jie et al., 2009). To improve ROS generation, noble metals, such as Ag and Au, have been considered to modify the TiO_2_ nanostructures. Recently, Park et al. verified hydrophilized TiO_2_ nanostructures could be a long-circulating SDT agent for treating both deep and superficial tumors (You et al., 2016). It has been well demonstrated that noble metals/TiO_2_ heterostructures not only exhibit an increased absorption activity in the visible-light region *via* local surface plasmon resonance (LSPR), but also prevent electron-hole recombination by trapping the photoexcited electrons (Ola and Maroto-Valer, 2015; Qu et al., 2022). Although, Ag nanoparticle modified TiO_2_ nanotubes (TNTs) display excellent antibacterial activity (Chen et al., 2017), the antibacterial performance would decrease with the solubilization of Ag in an aqueous environment. Owing to their excellent biocompatibility and chemical stability, Au nanoparticles (AuNPs) are extensively used in biosensors, photocatalysis, cancer therapeutics, and tumor imaging (Zhang S. et al., 2019; Xu J. et al., 2020; Zhao et al., 2022). Specially, AuNPs are ideal metal nanostructures because of tunable optical properties related to their geometrical structure (Wang et al., 2020a). In our previous study, the absorption of TNTs shifted from the UV region to the visible-light region by decorating with AuNPs (Zheng et al., 2020). Although Au/TiO_2_ interfaces have exhibited good performance in antibacterial PDT, the tissue penetration depth is still limited, endowing failure in inhibition and killing activities against pathogenic biofilms. Till now, less attention has been paid to the utilization of AuNPs-TNTs as an antibacterial platform in SDT, especially for the treatment of peri-implantitis.

In this study, we established an efficient, strongly penetrating, and safe nano-therapy platform and systematically evaluated the effect of AuNP loading on the antibacterial properties of TNTs under ultrasonic irradiation. On one side, the Au in AuNPs- TNTs could increase the generation of ROS by preventing the fast recombination of triggered electron-hole. For another, the ultrasound increased the depth of treatment. In addition, the nanoplatform exhibited a high security. In the evaluation of antibacterial activity, the major pathogen in oral environment (*P. gingivalis*), which is responsible for the failure and infections of implant, were chosen as the model bacteria. The antibacterial ability of the AuNPs- TNTs synthetic materials was found to rely on the Au loading amount. Based on all the above-mentioned results, AuNPs- TNTs were found to be a biocompatible platform with excellent ROS generating characteristics for an efficient antibacterial treatment. This study provides a new route to treat the inflammatory damage from bacterial biofilm on artificial dental implants.

## 2 Experimental and methods

### 2.1 Synthesis of TNTs arrays

The TNTs layer was fabricated using a two-electrode-setup anodic oxidation process following a previous study. In brief, Ti sheets were precleaned ultrasonically in acetone and ethanol for 20 min, rinsed three times with distilled water and dried in a N_2_ stream. Then, the working electrode and flakes were prepared by adding 0.27 M of NH_4_F in an electrolyte mixture of water/glycerol (50:50) and treated at 30 V for 4 h. A platinum foil was functioned as counter electrode. Eventually, the as-fabricated amorphous structured TNTs were crystallized into an anatase structure by annealing samples at 450°C in ambient air for 1 h (Wang G. et al., 2018).

### 2.2 Preparation of AuNPs-TNTs

All samples applied to AuNP decoration had anatase crystalline phases. For AuNPs-TNTs, all the samples were firstly immersed in the HAuCl_4_ solution (10 mg/ml) with pH of 9–10 for 16 h followed by rinsing with DI water carefully. Then, the samples were beamed with UV light (500 W) using a medium-pressure Hg lamp (maximum emission center around 360 nm); 8 cm for 30 min was the fixed distance between the lamps and samples (Gao et al., 2012). The above procedures were repeated three times in order to embellish a higher amount of AuNPs. Four groups of antibacterial surfaces were prepared as follows: 1) bare TNTs group (denoted as “TNTs”), 2) a low mount of AuNP-loaded TNTs which was prepared by one cycle of Au coating procedure (denoted as L-AuNPs-TNTs), 3) a medium mount of AuNP-loaded TNTs, which was prepared by two cycles of Au coating procedure (denoted as M-AuNPs-TNTs), 4) a high mount of AuNP-loaded TNTs, which was prepared by three cycles of Au coating procedure (denoted as H-AuNPs-TNTs).

### 2.3 Sample characterization

With the aid of field-emission scanning electron microscopy (FE-SEM, S-4800; Hitachi, Japan), the sample morphology was characterized. The structure of the samples was identified by use of an X-ray diffractometer (XRD, Rigaku, Tokyo, Japan) with Cu Kα radiation. Utilizing X-ray photoelectron spectroscopy (XPS, ESCA Lab 250, Thermo Fisher Scientific, New York, United States), the elemental composition was determined. The absorption range was registered using a UV-vis spectrophotometer (Shimadzu Corporation, Tokyo, Japan).

### 2.4 ROS capture experiments

2,2′-Bis(anthracene-9,10-diylbis (methylene))-dimalonic acid (ABDA) was selected to be a specific probe to detect the generation of ^1^O_2_. Typically, 5 µl of ABDA [10 µM in dimethyl sulfoxide (DMSO)] was put into AuNPs-TNTs solutions (200 µg/ml). Subsequently, the UV-vis absorbance range was registered using a Shimadzu UV-2550 spectrophotometer for the duration of ultrasound exposure (1.0 MHz, 1.5 W/cm^2^). The peak of appearance for ABDA at 378 nm indicates the generation of ROS (Liu et al., 2019).

### 2.5 Bacteria culture

*P. gingivalis* (ATCC33277, Manassas, VA, United States) was applied to produce a single-species biofilm. The bacteria were authorized by the Institutional Review Board of Jilin University, School of Dentistry and anaerobically cultured at 37°C in tryptic soy broth (TSB) added menadione (1 mg/L), L-cysteine hydrochloride (0.5 g/L), hemin (5 mg/L), and yeast extract (5 g/L) with 80% N2, 10% CO_2_ and10% H_2_ (Park et al., 2016). The inoculum was altered to 108 colony-forming unit counts (CFU/ml) for biofilm formation according to the standard curve of OD_600nm_ vs CFU/mL. The sheets were diverted to the 24-well plate, and every single well was inoculated in an anaerobic environment at 37°C. At every 24 h, the medium was changed to a fresh medium. Culturing for 4-day in total was adequate for the formation of a mature *P. gingivalis* biofilm (Cai et al., 2014).

### 2.6 *In vitro* antibacterial effect

An STYO9-PI assay was performed for the sterilization of *P. gingivalis* on the biofilm. After dealing with each group, SYTO9 (0.25 μM, Molecular Probes, Eugene, OR, United States)/propidium iodide (PI, 0.25 μM, Molecular Probes, Eugene, OR, United States) was added to the biofilm for staining live/dead bacteria, respectively. The red-stained bacteria represent the lifeless bacteria, and the bacteria colored by green represent the live bacteria. The images were obtained by using a confocal laser scanning microscope (CLSM, C2si, Nikon, Japan) (Zeng et al., 2017).

For CFU enumerating, the 4-day biofilm was diverted into vials with 1 ml media, scraping, sonicating, and vortexing (Thermo Scientific Fisher) to obtain the biofilms. In the wake of a serial attenuation, the bacterial suspension was dispersed onto the TSB culture medium and anaerobically incubated at the temperature of 37°C for 72 h. The number of colonies was counted utilizing a colony counter and CFU was calculated utilizing a dilution factor. In order to evaluate the activity of metabolization of *P. gingivalis* bio-membranes, an MTT method was performed. Handle the Ti sheets with biofilms by MTT dye and culture at the temperature of 37°C for 1 h, followed by treating equivalently with DMSO, shaken in horizontal direction for 30 min in the darkness. Pipet the solution into a new 96-well plate, and then measure the absorbance at OD_540nm_. Calculate the metabolic activity of the biofilm by the higher absorbance (Li et al., 2015). All of the samples were assayed in 3 replicates.

### 2.7 Cell culture

The human gingival fibroblasts (HGFs) were cultivated in the cell culture media, containing 89% Dulbecco’s modified Eagle’s medium (DMEM), 10% (v/v) fetal bovine serum (FBS), and 1% antibiotics (penicillin and streptomycin) at 37°C in a damp atmosphere with 5% CO_2_ (Tsugeno et al., 2014).

The biosecurity was evaluated with cytological staining. The nucleus of HGFs was stained using 4,6-diamidino-2-phenylindole (DAPI), while the cytoplasm was dyed using fluorescein isothiocyanate (FITC). Then, the CLSM images were recorded. To study the cytotoxicity of those slices with different samples, HGFs inoculated in 24-well plates at a density of 2.5×10^5^ per well were incubated with TNTs, L-AuNPs-TNTs, M-AuNPs-TNTs, and H-AuNPs-TNTs for 24 h. Relative cyto-activity was estimated in virtue of a cell counting kit-8 (CCK8) assay (Wang et al., 2016).

Fresh rabbit blood was detached from plasma by means of centrifuging at 1500 rpm for 15 min at 4°C. The separated RBCs were first washed with PBS to make the supernatant translucent, and then suspended again in PBS. Then, TNTs slices with different treatments were added to the RBC turbid liquid. As long as putting in an incubator at the temperature of 37°C for 2 h, the suspension was separated at the speed of 1500 rpm. The supernatant obtained from every single centrifugal cuvette was applied to assay the hemoglobin release utilizing a microplate reader at OD_576 nm_ (Mazare et al., 2016). Comparison groups were detected under the identical condition; PBS was added as the negative control. In contrast, Triton X-100 at 0.5% was added to be the positive comparison group. Each group used six samples. Use the following formula to calculate the percentage of hemolysis:

$$Hemolysis=\frac{\left({OD}_{sample}-{OD}_{negative\;control}\right)}{\left({OD}_{Positive\;control}-{OD}_{negative\;control}\right)*100\%}.$$ (1)

### 2.8 Statistical analysis

The data were represented as the means ± standard error of the mean. The significant differences between sample groups were performed by a one-way analysis of variance with a Tukey’s post hoc test using the GraphPad Prism 7.0. A value of *p* < 0.05 was considered to indicate statistical significance.

## 3 Results and discussion

### 3.1 Material design and morphology characterization

**FIGURE 1:**
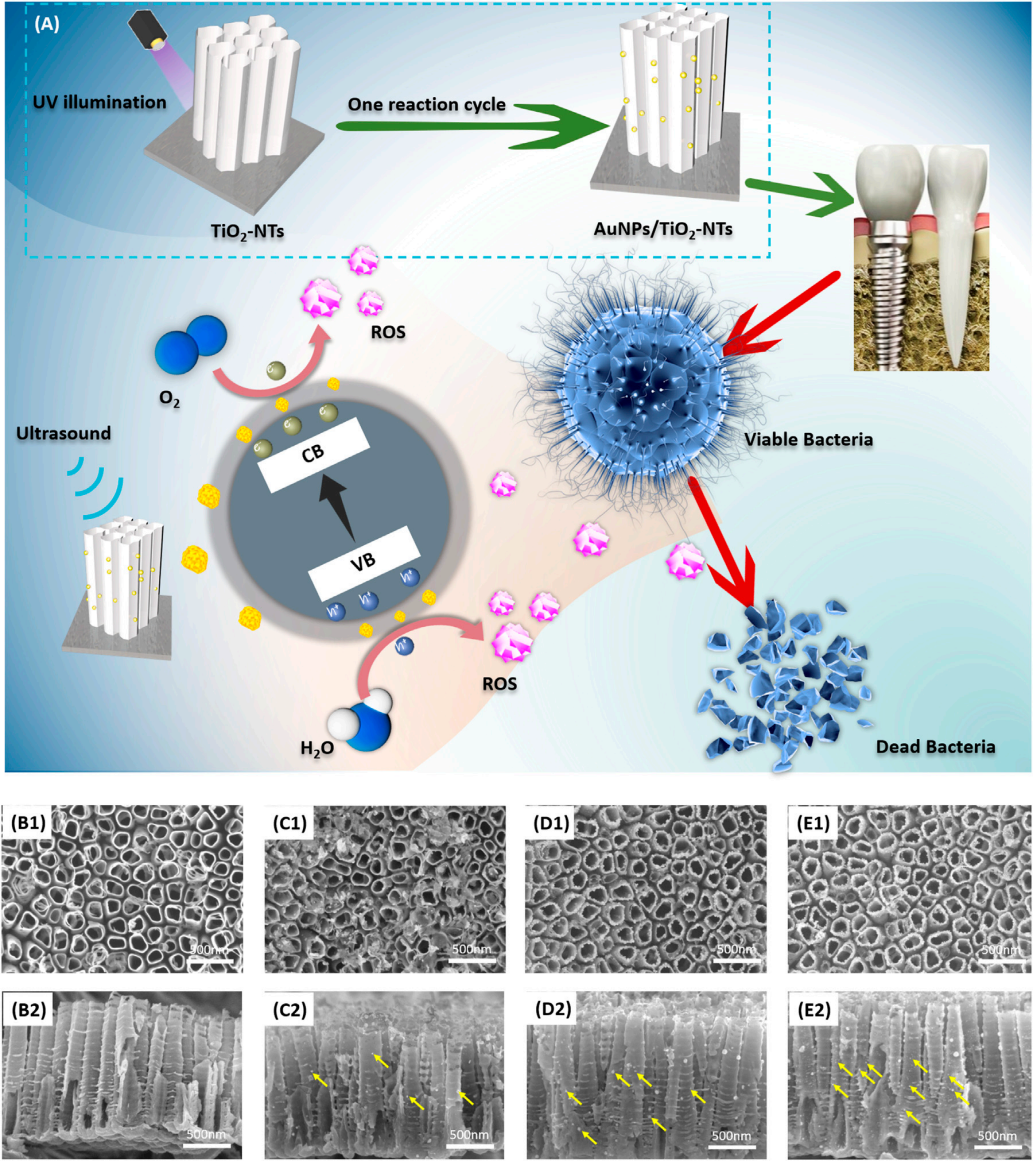
**(A)** Schematic image of TNTs surface with AuNP decoration. This designed surface exhibited antibacterial activity after ultrasound irradiation. The vertical view **(B1–E1)** and cross-sectional view **(B2–E2)** of **(B)** TNTs, **(C)** L-AuNPs-TNTs, **(D)** M-AuNPs-TNTs, and **(E)** H-AuNPs-TNTs. Yellow arrows show AuNPs loaded on the tube wall.

As the schematic image shown in Figure 1A, TNTs with AuNPs decoration were designed and fabricated for potential application in dental implants. The functional surface modification of implant was achieved by ultrasound irradiation and subsequent release of ROS to induce the lipid peroxidation of bacteria membrane. The bacteria were dissolved, broken, and killed owing to the excellent antibacterial activity of highly activated ROS.

Figure 1(B1–E1) show the SEM images of TNTs and AuNPs-TNTs. TNTs exhibited a self-standing nanotubular structure with a diameter in the range of 150 ± 10 nm (Figure 1B1) and around 1.3 μm in length (Figure 1B2). Before the AuNP embellishment, in order to transfer the amorphous TiO_2_ to the anatase phase, the TNTs were annealed at 450°C in air and lasted for 1 h. In the time of exposing AuCl_4_^−^-TNTs to UV light, the photo-induced electrons were triggered to the conduction band of TiO_2_. Subsequently, these electrons drive to the surface of TNTs and then reduces the combination between the adsorbed AuCl_4_^−^ and Au (0) by the internal field. In addition, from dental materials’ perspective, such an engineered cover with TNTs ranging from 55 to 110 nm would not impact the mechanical properties when used as dental implant base, demonstrating its beneficial fracture load-bearing capabilities under external masticatory pressure conditions. Consequently, this engineered design did not have mechanical compromise and it is a promising dental implant surface.

Furthermore, three categories of AuNPs-TNTs had uniform tube diameters, which indicated that Au decoration would not compromise the structure of TNTs (Figure 1C1–E1). AuNPs with good morphology were observed on the tube surface with a shape of 20 nm in contrast with the bare TNTs.

As shown in Figure 1(B2–D2), the images of intersecting surface show that the tube lengths of all samples were similar, and AuNPs were grafted on the tube wall. According to an increase in the reaction cycles, the AuNPs were located deeper into the tubular structure, which revealed that SPR-Au improved the potential of photocatalytic. Predictably, it led to an increase in nanoparticle density when the number of modification cycles was in the high level (Figure 1C1–E1, C2-E2). These AuNPs are equally distributed on the inner and outer surfaces of nanotubes. Notably, the increase of decoration cycles did not instigate a segregation phenomenon. The size of these nanoparticles was similar to the size obtained in the first cycle.

### 3.2 Physical and chemical characterization of AuNPs loaded on TNTs

**FIGURE 2:**
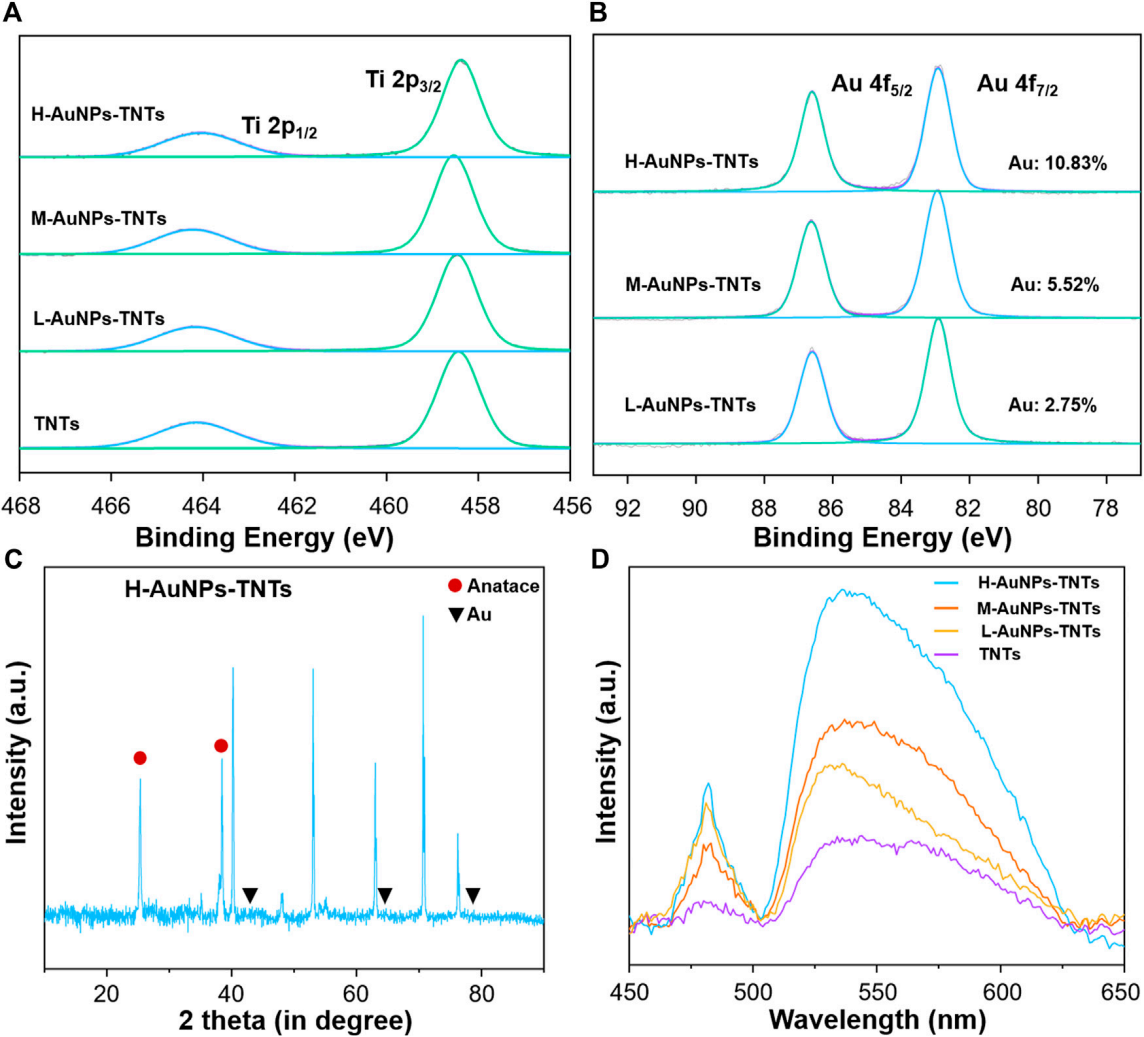
**(A)** XPS measurement of TNTs and AuNPs-TNTs substrate. **(B)** XPS measurement of Au 4f spectra for AuNPs-TNTs. **(C)** XRD patterns of H-AuNPs-TNTs. **(D)** UV-vis spectra of pure TNTs and AuNPs-TNTs with different cycles.

We carried out XPS analysis as a means to further validate the chemical composition of the as-prepared TNTs and AuNPs-TNTs surface. The survey and high-resolution XPS spectra of AuNPs- TNTs are shown in Figure 2A,B and Supplementary Figure S1. After UV-induced photoreduction, the emergence of Au 4f signals was observed. The intense peaks at 83.0 and 86.8 eV could be observed in the Au 4f spectrum in Figure 2B, which indicated a normal state of Au 2p and the metallic Au generation in AuNPs-TNTs. In addition, the results of XPS analysis indicate that the Au contents Au in different AuNPs-TNTs varied. Draw a distinction between L-AuNPs-TNTs and M-AuNPs-TNTs, H-AuNPs-TNTs had the highest amount of Au, and the surface loading mount is up to 10.83%. For comparison, the Au content in the L-AuNPs-TNTs group is 5.52% and in M-AuNPs-TNTs group is 2.75%. An X-ray diffraction (XRD) analysis was also conducted to designate the TNTs before and after decoration with AuNPs by photo-catalytically induced reduction (Figure 2C and Supplementary Figure S2). Beside the typical peaks obtained from the anatase crystalline state of TiO_2_, the diffraction peaks at θ = 45, 64, and 77° are correlated to the monoclinic Au phase, and detected on the obtained sample (JCPDS No: 01–1174). The increasing quantity of AuNP incorporation was verified from the UV-vis spectra. As shown Figure 2D, an absorption peak representing the Au appeared in the 500–700 nm range, and it showed a growing trend with the Au-loading amount, especially on the H-AuNPs-TNTs sample.

As surface wettability is an important required property for biomedical artificial implants, the contact angle (CA) of TNTs and AuNPs-TNTs samples was measured by a contact angle system (3 μl, room temperature, Dataphysics-OCA20, Germany) (Supplementary Figure S3). Owing to the presence of abundant Ti-OH groups on the surface, the as-formed TNTs are hydrophilic (CA = 11.8). Although the contact angles slightly increased with AuNP coating amount, the surface still maintains hydrophilicity even at a high AuNPs loading, i.e., the contact angles of L-AuNPs-TNTs, M-AuNPs-TNTs, and H-AuNPs-TNTs were determined as 26.5, 26.9, and 28.2, respectively.

### 3.3 ROS generation under ultrasound

**FIGURE 3:**
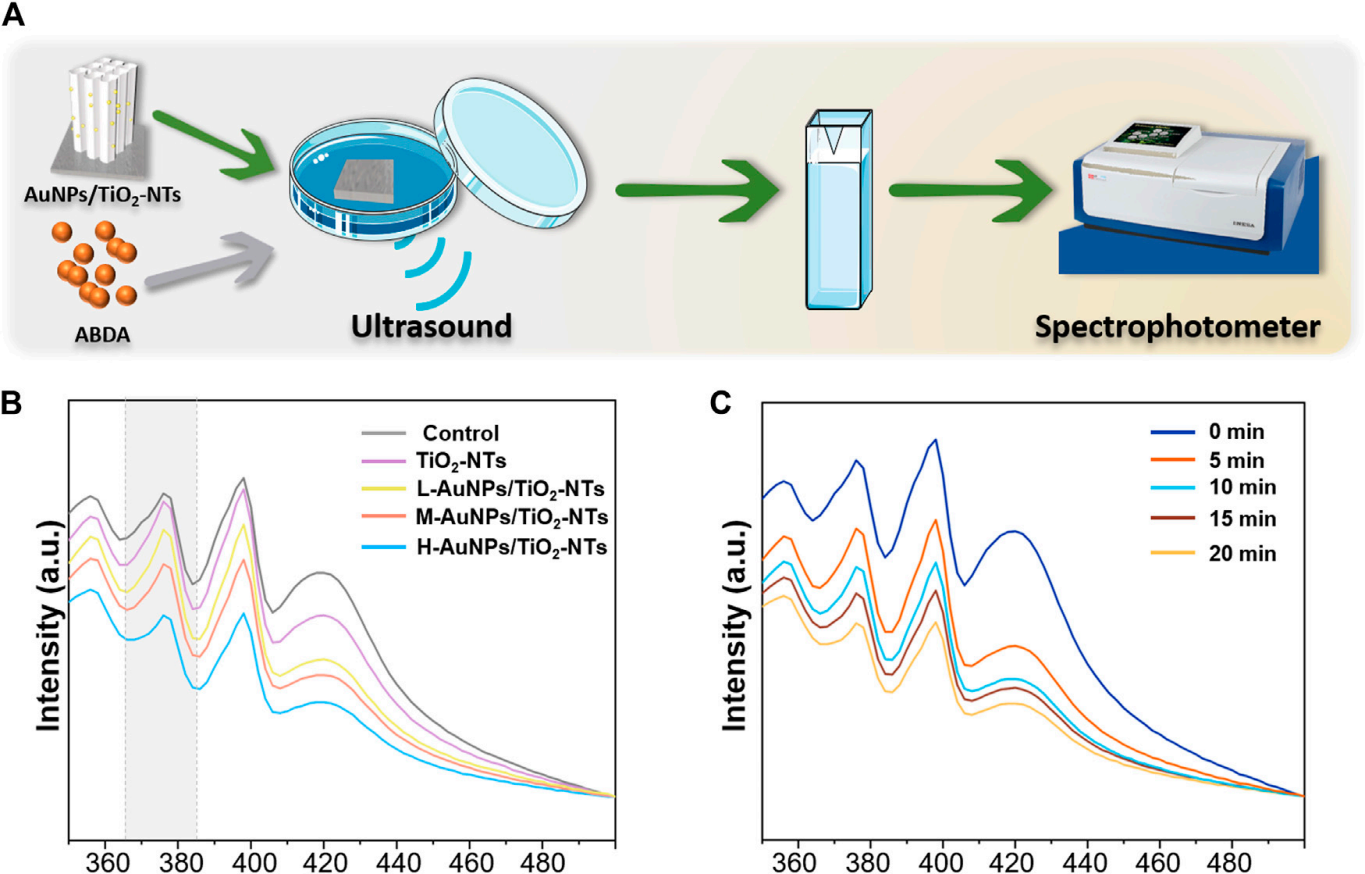
**(A)** Schematic images of ^1^O_2_ generation with time. **(B)** ROS generation testified by the absorbance of ABDA under ultrasound irradiation for 10 min. **(C)** The amount of ROS generation changed in the H-AuNPs-TNTs group under ultrasound treatment in 20 min.

To achieve a satisfactory antibacterial outcome, a high-caliber of ROS is preferred. The ROS can induce MDA, resulting in membrane lipid solubilization, which further kills the bacteria (Yang et al., 2019). Therefore, a dynamic ROS generation/production yield is crucial for achieving a high antibacterial efficiency. In general, ^1^O_2_ has been previously reported as the main ROS species generated during ultrasound treatment (Zhang et al., 2021). To gain insight on the antibacterial possibility, the ^1^O_2_ generation kinetics on the different AuNPs-TNTs groups was evaluated using ABDA as the trapping agent, and the results were obtained using a UV-vis spectrophotometer (Figure 3A).

ABDA is widely used as a trap for ^1^O_2_ in the biomedical realm, and also it could be easily monitored by UV-vis. The underlying operational principle involves the disappearance of its main absorption centered at 375 nm after reaction with ^1^O_2_, which could be recorded to generate ROS (Cao et al., 2019).

As shown in Figure 3B, the ROS production on L-AuNPs-TNTs and M-AuNPs-TNTs was higher than that of TNTs. Obviously, the absorbance of ABDA at 378 nm showed the most significant decreasing tendency due to the consumption of ROS, indicating that H-AuNPs-TNTs generated the maximum ^1^O_2_ dose. This performance indicates that the quantity of ROS for AuNPs-TNTs was positively correlated with Au content decoration. This is probably because the band gap of AuNPs-TNTs (2.90 eV) was narrower than absolute TiO_2_ (3.20 eV) (Chen et al., 2011), preventing the rapid recombination between excited electrons and holes, and leading to a higher quantum yield of ROS production (Felip-León et al., 2019). it could also speed up the rate constant and thus increase the productivity of ROS when combined Au with TiO_2_ (Chen et al., 2011). To evaluate the ROS production kinetics, the time-dependent ABDA consumption in the presence of H-AuNPs-TNTs under ultrasound treatment in each 5 min time interval was plotted in Figure 3C. The change in ABDA absorption indicates the continuous and controllable ^1^O_2_ generation under sonodynamic stimulation. These results show that ultrasound activation is an effective route to release negligible ROS on TiO_2_-based nanomaterials instead of applying the photocatalysis. Based on ^1^O_2_ generation, it can be hypothesized that AuNPs-TNTs would be a promising material to kill the pathogen by ultrasound activation. The time-sequential and rational function of H-AuNPs-TNTs may enhance the antibacterial application of TNTs in infectious disease. The morphologies of H-AuNPs-TNTs before and after the ultrasound irradiation were evaluated (Supplementary Figure S4). The sample maintained its initial structure with intact nanotubes and the well-decorated AuNPs in ultrasound, verifying the excellent mechanical stability of AuNPs-TNTs. Plenty of ROS generation and good stability of AuNPs-TNTs by ultrasound treatment indicate good potential of the as-proposed AuNPs-TNTs as an STD agent in antibacterial application.

### 3.4 Antibacterial properties of AuNPs-TNTs samples

**FIGURE 4:**
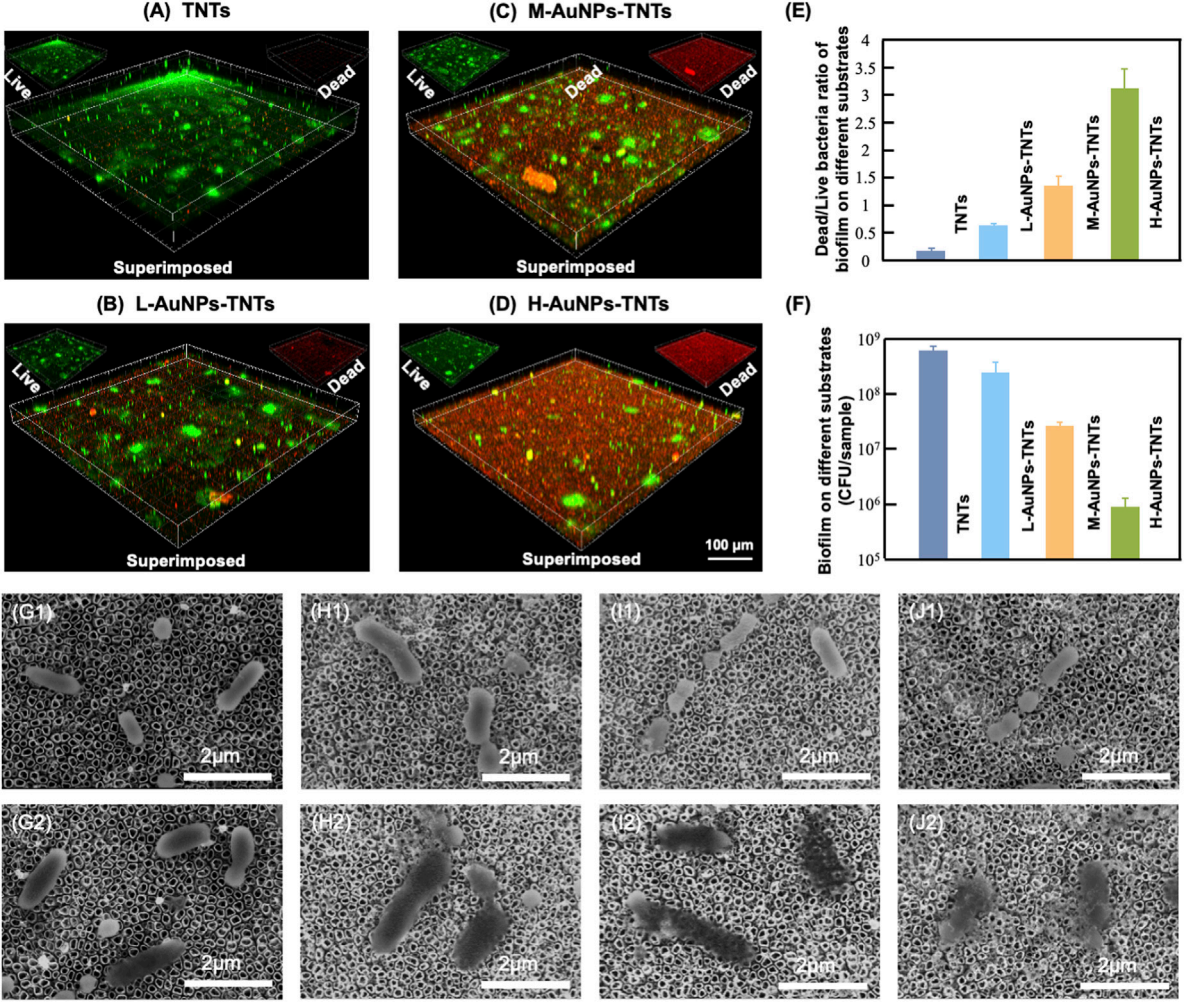
Antibacterial effect. **(A–D)** Confocal fluorescence microscopy images of *P. gingivalis* incubated with AuNPs-TNTs at ultrasound power densities 1.5 W/cm^2^ and stained with STYO9-PI (green: live bacteria, red: dead bacteria). **(E)** Colony formation units of *P. gingivalis* in different agar plates. **(F)** Statistical bar chart of dead/live bacteria. SEM images show the morphology of *P. gingivalis* adhered on **(G)** TNTs, **(H)** L-AuNPs-TNTs, **(I)** M-AuNPs-TNTs, and **(J)** M-AuNPs-TNTs samples before **(G1–J1)** and after **(G2–J2)** ultrasound-induced antibacterial process.

The antibacterial properties were then studied to elucidate the peri-implantitis therapeutic mechanism of the AuNPs-TNTs samples. In this study, *P. gingivalis*, one of the major pathogenic bacteria that may occur in the peri-implantitis to form a biofilm *in vivo*, was selected as the representative pathogen. Figure 4(A–D) show the antibiofilm efficacy of different nanostructure *via* typical dead/live bacterial images. PI was used to stain dead bacteria and exhibited as red color bacteria by pseudo-color processing, styo6 was used to stain live bacteria and exhibited as green color bacteria by pseudo-color processing. Figure 4E shows the ultrasound inactivation results of *P. gingivalis* using the traditional colony counting method and agar plates. Compared with the TNTs group, the CFU values for M-AuNPs-TNTs group, L-AuNPs-TNTs group, and H-AuNPs-TNTs group exhibited a reduction of approximately 1, 2, and 3 orders of magnitude for periodontal biofilm, respectively. The difference in bactericidal abilities is associated with different ROS yields on these samples during the ultrasound treatment. In this case, ROS, a strong oxidant, had the characteristic of high reactivity, causing various ROS-related reactions, such as oxidation, reduction, or dissolution. These reactions further cause bacterial lipid oxidation and membrane lipid destruction, leading to bacterial death. Remarkably, H-AuNPs-TNTs act as a ROS-generation warfare agent, exhibiting superior antibacterial properties among the tested groups. A statistical bar chart of dead/live bacteria is shown in Figure 4F, consistent with the fluorescent graphs in Figure 4(A–D).

The SEM images show the morphology of intact bacteria (Figure 4G1–J1) and the bacteria undergoing ultrasound treatment (Figure 4G2–J2) on different sample groups. *P. gingivalis* exhibited a smooth and unbroken cell body. In addition, no difference was observed for the bacteria morphology on these different samples before ultrasound induced antibacterial inactivation. The intracellular substance was released into the extracellular microenvironment following the perforation of cell membrane which can be confirmed by the bacterial fragments found nearby. However, the morphology of *P. gingivalis* adhered on AuNPs-TNTs samples showed remarkable changes after ultrasonication irradiation. The bacteria were deformed and collapsed. However, the bacteria loaded on TNTs still maintained most of their original structure even under the same ultrasound experiment. In this study, AuNP decoration facilitated the triggering of TNTs under ultrasound owing to the surface plasmon resonance (SPR) influence of precious metals. At the same time, the power density of an ultrasound source is 1.5 W/cm^2^, which could be easily attained from a conventional clinical device. This would overcome the limitation of light penetration. TNTs decorated with a great quantity of AuNPs had perfect inhibitory effect on the main pathogenic bio-membrane, which implied favorably antibacterial potential in terms of clinical applications. This nano-engineered structure showed parallel antibacterial efficiency with porphyrin sensitizer-DVDMS added TNTs and superior to TNTs only (Wang et al., 2020b).

### 3.5 *In vitro* cellular compatibility

In addition, ultrasound, a mechanical wave with better ability of tissue penetration and limited energy attenuation, is considered safer than the visible light in PDT (Zhang S. et al., 2019). Large amounts of ROS would be produced when activated by external ultrasound energy, resulting in an intense restriction and killing activities against plaque biofilm (Wang Z. et al., 2018). Nevertheless, with regard to the treatments of infectious diseases, excessive production of ROS could worsen the internal inflammation. Generally, the diffusion distance of ROS is just about 100 nm and the half-time is shorter than 0.04 μs (Misba et al., 2018), thereby, the distance that ROS diffuse into the cells is important for the SDT activity (Zhang T. et al., 2019). According to a contact killing mechanism, the modification of the surface AuNPs-TNTs in this research provided beneficial antibacterial activity. It is interesting that peri-implantitis is caused by a dental biofilm attached to implants. The ROS produced by ultrasound-photocatalytic process would cause the inactivation of the attached bacteria, thereby preventing and impeding the development and progress of peri-implantitis. Furthermore, in a typical ultrasound process, collapse cavitation can bring about temperatures in excess of 5000 K and over 50 MPa pressures on a nanosecond time scale (Merouani et al., 2014). These generate a high liquid shear force, impact waves, localized heating, and appearance of free substrate, contributing to the antibacterial activity of sonosensitizer *via* several mechanisms.

**FIGURE 5:**
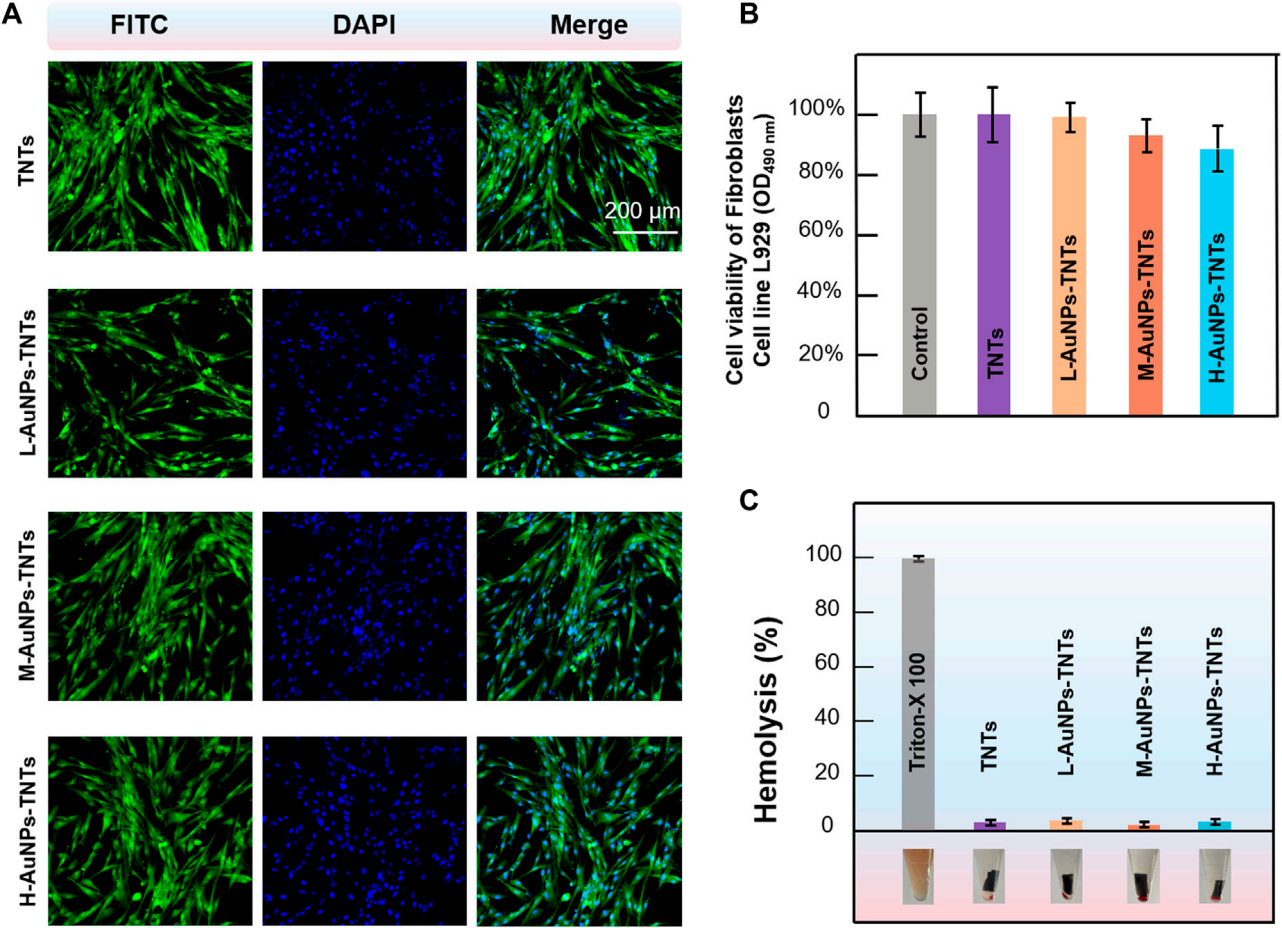
Confocal fluorescence microscopy images of HGFs (incubated with AuNPs-TNTs stained with DAPI and FITC, scale bar: 200 μm. **(B)** Viability of HGFs incubated with AuNPs-TNTs. **(C)** Hemolysis of RBCs coincubated with AuNPs-TNTs.

The biocompatibility of nanomaterials is a key indicator for biological applications. The initial interaction and reaction between cells and the surfaces of biomaterials decide the life span of an implants (Bowles and Setton, 2017). Insufficient cell adhesion hinders tissue repair and regeneration, resulting in implant instability, usually leading to the failure of dental implant and even inducing severe septicemia. To evaluate the feasibility of AuNPs-TNTs used as an antibacterial platform, cellular cytotoxicity of AuNPs-TNTs platform towards HGFs was investigated. The cytotoxicity of AuNPs-TNTs was evaluated based on the apoptosis and morphological changes of HGFs. As shown in Figure 5A, HGFs were separately incubated with TNTs, L-AuNPs-TNTs, M-AuNPs-TNTs, and H-AuNPs-TNTs, and then stained with DAPI and FITC. The nuclei which were labeled by DAPI in each group show blue fluorescence, homogeneous distribution, and intact structure. The FITC-labeled cytoplasm in each group emits green fluorescence, fusiform, and consecutive. These results indicate an ignorable cytotoxic effect from AuNPs-TNTs surface. To quantify the cytotoxicity of AuNPs-TNTs platform toward HGFs, we carried out a standard trial CCK-8 cell viability. After incubation with the three types of AuNPs-TNTs and TNTs samples for 24 h, the cell viability of HGFs was measured using the method of CCK-8. As shown in Figure 5B, the cell viability of the HGFs on these samples is still higher than 85%, indicating that the as-proposed AuNPs-TNTs antibacterial materials have low cytotoxicity and satisfactory biocompatibility. By further increasing the incubation time to 48 and 72 h, the HGFs still maintained good state on H-AuNPs-TNTs with ignorable apoptosis and morphologic changes (Supplementary Figure S5). Moreover, we also examined the influence of the test materials on the wholeness of RBC membrane that might lead to the fracture of RBC, and thus calculated the hemolytic index. The hemolysis of RBCs in the presence of TNTs, L-AuNPs-TNTs, M-AuNPs-TNTs, and H-AuNPs-TNTs was lower than 5%, indicating excellent hemocompatibility (Figure 5C).

These results indicate that the presence of AuNPs-TNTs had less negative impact on the proliferation and state of cell. These phenomena can be ascribed to the large surface area of nanotubular TiO_2_-based materials, which provide plenty of sites, with the features of better hydrophilicity and protein adsorption capacity. Due to these advantages, cell attachment and proliferation are improved, thereby promoting osseointegration (Su et al., 2018). Moreover, the negatively charged TNTs and AuNPs-TNTs surface can attract positively charged proteins, which would also improve cell attachment (Kabaso et al., 2011). As the result of high surface charge density, there is a strong bonding between the AuNPs-TNTs and cells (Su et al., 2018). In addition, noble-metal nanoparticles would also improve cell proliferation *via* enhancing the gene expression (Balu et al., 2020).

Furthermore, *in vivo* animal study was conducted to confirm the wound tissue healing around different surfaces. As shown in Supplementary Figure S6, substantial inflammatory cells infiltrated around the four groups, indicating local inflammatory reaction was induced by LPS. While, both H&E staining images and the histomorphometric analysis demonstrated that the total number of inflammatory cells in three groups of AuNPs/TNTs were reduced significantly than TNTs group. The thick collagen fibers (Masson staining) of the three groups of AuNPs/TNTs were significantly increased compared with TNTs group, fibroblasts tend to be mature. Whereas, the TNTs group exhibited blood clots and large amount of big and round fibroblasts, demonstrating that they were still at the early stage of wound healing.

## 4 Conclusion

In conclusion, we designed a TNTs substrate decorated by AuNPs for application prospect in the antibacterial effect of dental implant surfaces. AuNPs decoration on TNTs surface would induce a large amount of ROS generation just by applying a simple ultrasound treatment. The generated ROS has been demonstrated to be effective to inactivate the bacteria attached on the sample surface. Importantly, the results of cytotoxicity tests show that AuNPs-TNTs have excellent biocompatibility for promising implant applications. Considering securer and superior tissue penetration accompanied by limited energy attenuation than photo-catalytically triggered bacteria inactivation, the AuNPs-TNTs provide a promising surface for achieving excellent antibacterial properties against the main pathogenic biofilm by SDT. Our study thus proposes a strategy to develop a nanoplatform for effective anti-bacteria combining ultrasound with TiO_2_ doping metal ions, which may extend the applications of TiO_2_-based nanomaterials in treating the inflammatory damage from bacterial biofilm on artificial dental implants Park et al., 2016, Xu et al., 2020.

## Data Availability

The original contributions presented in the study are included in the article/Supplementary Material, further inquiries can be directed to the corresponding author.
