# Supplementary material for: Gold nanoparticle decoration potentiate the antibacterial enhancement of TiO2 nanotubes via sonodynamic therapy against peri-implant infections
Source: Front Bioeng Biotechnol. 2022 Nov 17;10:1074083. doi: 10.3389/fbioe.2022.1074083 (PMC9713247; doi:10.3389/fbioe.2022.1074083)
Supplement: Supplementary file 1 [file Table1.DOCX]

Supporting Information

# Gold Nanoparticle Decoration Potentiate the Antibacterial Enhancement of TiO_2_ Nanotubes via Sonodynamic Therapy against Peri-implant Infections

**Yue Sun^1,3#^, Wenzhou Xu^2#^, Cong Jiang^1^, Tianyu Zhou^1^, Qiqi Wang^2^, Lan A^1,3^***

*^1^Department of Oral Implantology, School and Hospital of Stomatology, Jilin University, Changchun 130021, China*

*^2^Department of Periodontology, School and Hospital of Stomatology, Jilin University, Changchun 130021, China*

*^3^Jilin Provincial Key Laboratory of Sciences and Technology for Stomatology Nanoengineering, Changchun 130021, China*

** To whom correspondence should be addressed: E-mail address: hialan1983-2001@jlu.edu.cn*

*# These authors contribute equally to this work*

Experimental Section

**Materials**

Ti sheets (grade IV, 0.1 mm thick) were purchased from Baoji Titanium Industry Co., Ltd. (Baoji, Shaanxi, China). Acetone and ethanol were purchased from Sinopharm China. NH_4_F were purchased from Sinopharm China. HAuCl4 solution (10 mg mL^−1^, pH 9–10) were purchased from Sinopharm China. 2,2’-bis (anthracene-9,10-diylbis (methylene))-dimalonic acid (ABDA) was purchased from J&K Scientific Ltd. (San Jose, USA). DMSO was purchased from Sigma-Aldrich China. *Porphyromonas gingivalis* (ATCC33277) was purchased from Manassas, VA, USA. Human gingival fibroblasts (HGFs) were purchased from ScienCell (San Diego, USA) Fetal bovine serum (FBS) was purchased from Sigma-Aldrich China.Tryptic soy broth (TSB) was purchased from Sigma-Aldrich China. Menadione, L-cysteine hydrochloride, yeast extract and hemin were purchased from Sigma-Aldrich. SYTO9 /PI was purchased from Molecular Probes, Inc. Eugene, OR, USA. 4,6-diamidino-2-phenylindole (DAPI) and fluorescein isothiocyanate (FITC) were purchased from Millipore Sigma. Methyl thiazolyl tetrazolium (MTT), (3-Mercaptopropyl) trimethoxysilane (MPTMS) and 4-Mercaptophenylboronic acid (MPBA) were obtained from Aladdin China. All the reagents were used as received without any purification and the water used in all experiments was deionized (DI) water (>18 MΩ cm).

**Instruments:**

The morphologies were characterized using a scanning electron microscopy (SEM Hitachi S-4800 Japan). X-ray diffractions were acquired on an X-ray diffraction spectrometer (Philips X'pert-MPD PW3040, Netherlands). X-ray photoelectron spectra (XPS) was recorded on a Perkin–Elmer Physical Electronics 5600 spectrometer. UV-vis absorbance spectra was detected by a Shimadzu UV-2550 spectrophotometer (Shimadzu Corporation, Tokyo, Japan) Fluorescence spectra (F7000, Hitachi, Japan) were used in the DPBF assays and RDPP assays respectively. The live/dead bacteria images were captured by confocal laser scanning microscope (CLSM, C2si, Nikon, Japan) The number of colonies was counted by a colony counter (Reichert Inc., Depew, NY, USA). MTT assays were tested by a microplate reader (Gen5, BioTek, USA). All the cell images were taken by confocal laser scanning microscopy (FV1200, Olympus, Japan). The structure of the samples was identified using an X-ray Diffractometer (Philips X'pert-MPD PW3040) with Cu Kα radiation.

**Animal Wound Healing Test.** The experiment was followed a protocol approved by Jilin University (JLUKQ #SY2020011003). Male Kunming mice at 6-8 weeks were divided into TNTs, L-AuNPs-TNTs, M-AuNPs-TNTs and H-AuNPs-TNTs groups.

After anesthesia, the back skin of the mouse was made a circular incision of approximately 4 mm diameter and injected with 10 µg LPS to build the inflammatory model. The wounds were treated as the design. Mice were sacrificed at the 8^th^ day and skin tissue samples were excised. The skin tissue samples were fixed by paraformaldehyde and embedded in paraffin for the preparation of histological sections. After being stained with HE and Masson, the samples were examined with microscope (Olympus).


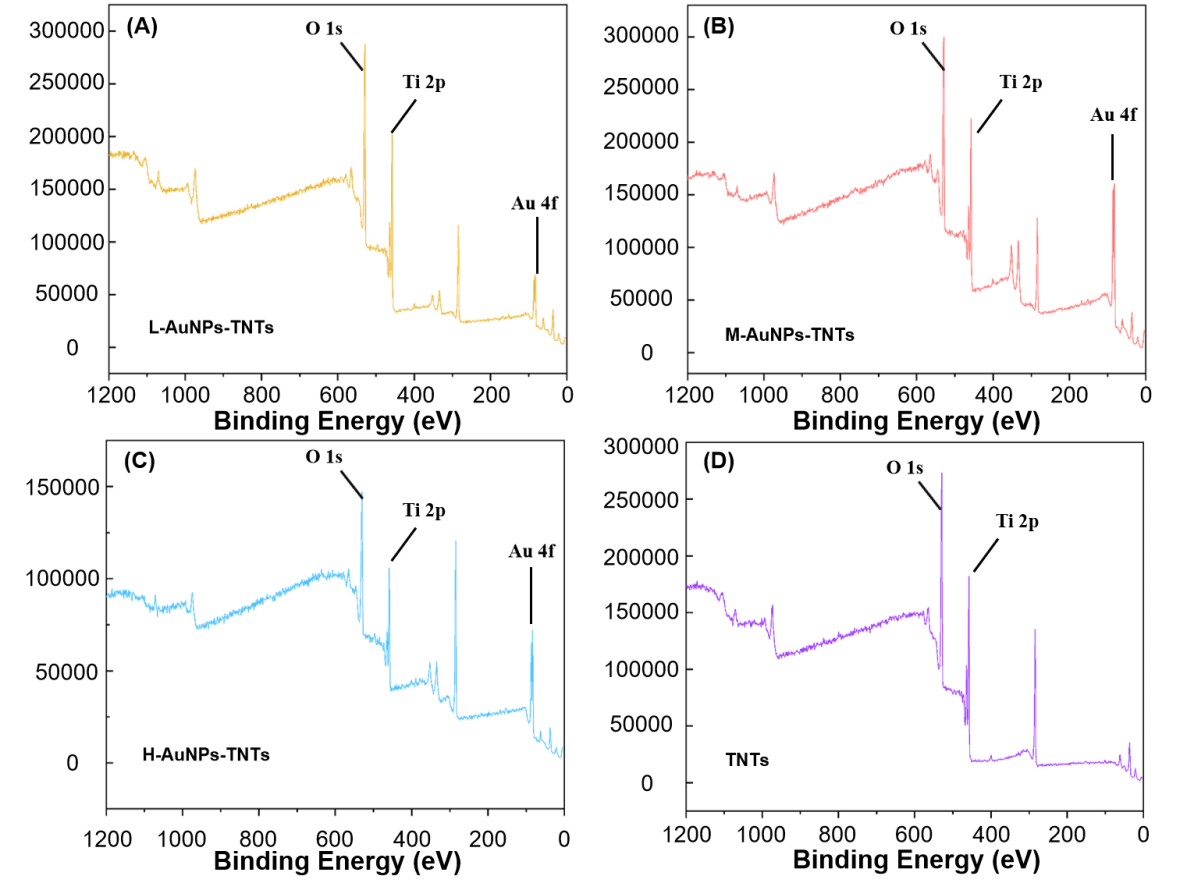


**Figure S1** (A-D) XPS survey spectra of TNT substrate and AuNPs-TNTs.


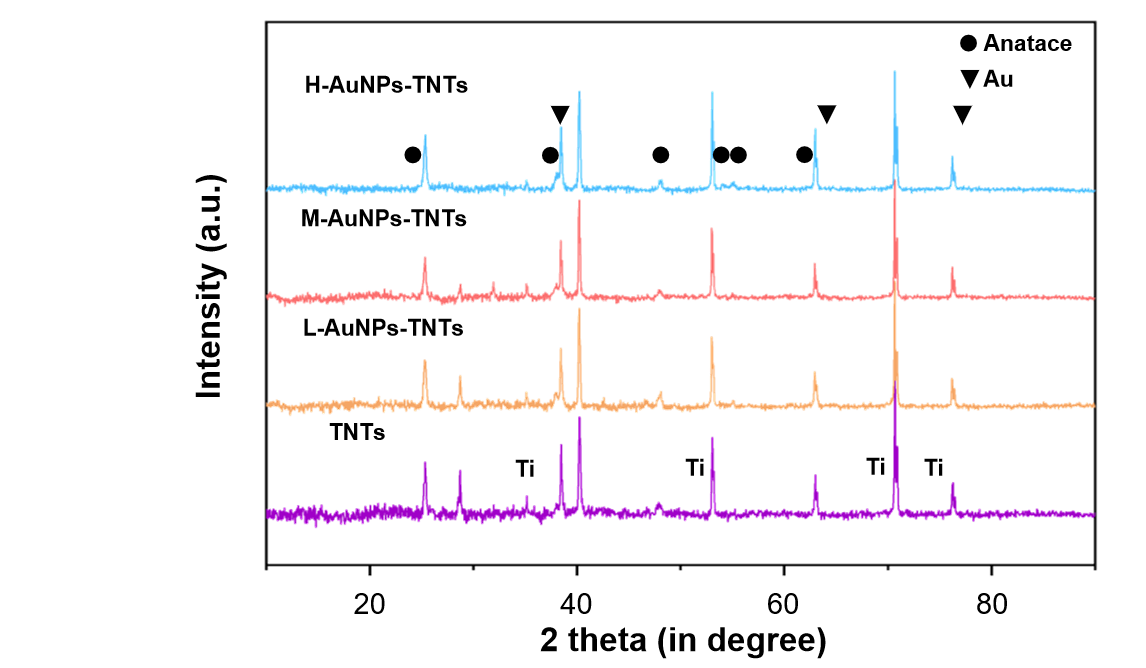


**Figure S2** XRD patterns of H-AuNPs-TNTs, M-AuNPs-TNTs, L-AuNPs-TNTs, and TNTs.


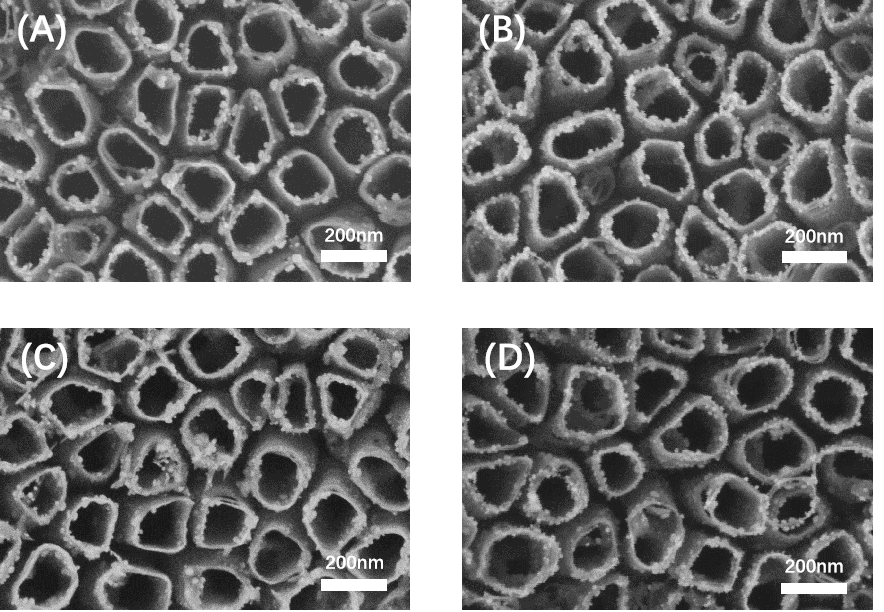


**Figure S3** SEM of H-AuNPs-TNTs (A) before and after ultrasound (70W)treating for (B) 1 min, (C) 2 min, and (D) 3 min.


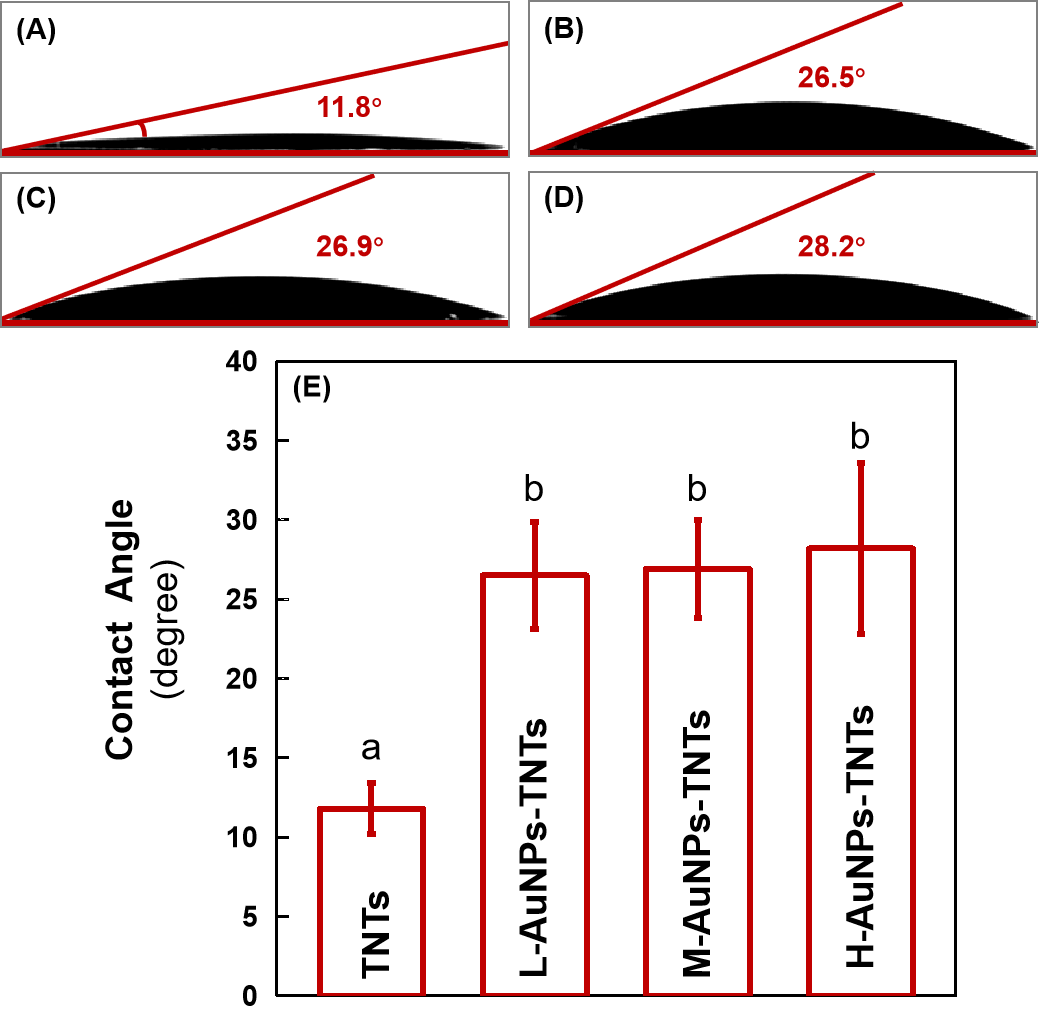


**Figure S4** Optical images of a water droplet on (A) TNTs, (B) L-AuNPs-TNTs, (C) M-AuNPs-TNTs, (D) H-AuNPs-TNTs, and (E) histograms of contact angles.


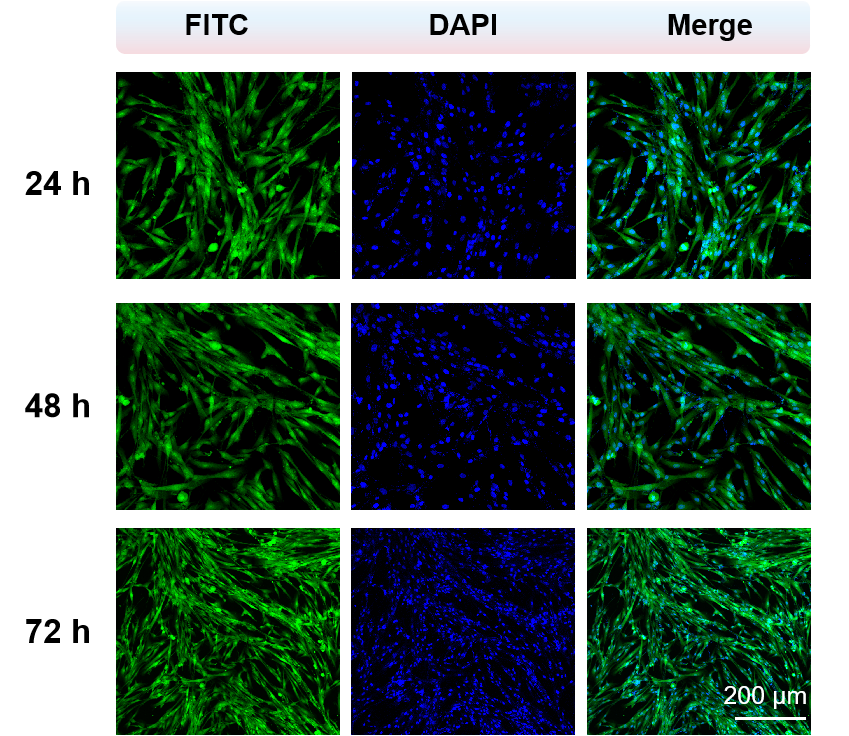


**Figure S5** Confocal fluorescence microscopy images of HGFs stained with DAPI and FITC at different incubation time periods on H-AuNPs-TNTs (scale bar: 200 μm.)


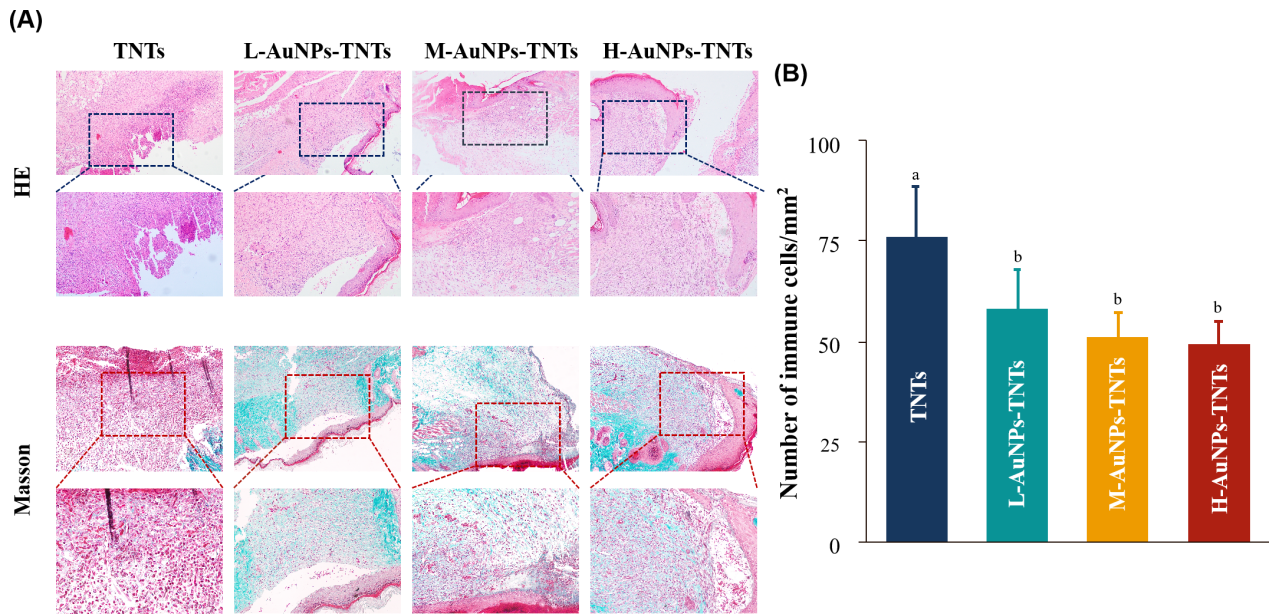


Figure S6 (A) HE and Masson staining images. HE staining showed that the inflammatory cells of L-AuNPs-TNTs, M-AuNPs-TNTs, H-AuNPs-TNTs groups were reduced significantly than TNTs group. Collagens stained blue were observed in the Masson staining images. The thick collagen fibers of L-AuNPs-TNTs, M-AuNPs-TNTs, H-AuNPs-TNTs groups were significantly increased, compared with TNTs group, fibroblasts tend to mature. Whereas, the TNTs groups exhibited blood clots and amount of big and round fibroblasts, and were still in the early stages of wound healing. (H) The corresponding quantification of immune cells in H&E staining sections. (n = 5, p < 0.05)

**References**

Gao Z.D., Liu H.F., Li C.Y., Song Y.Y. (2012). Biotemplated synthesis of Au nanoparticles-TiO_2_ nanotube junctions for enhanced direct electrochemistry of heme proteins. *Chem. Commun.* 49 (8), 774-776. [doi:10.1039/c2cc38183d](https://doi.org/10.1039/c2cc38183d)

Park S.W., Lee D., Choi Y.S., Jeon H.B., Lee C.H., and Moon J.H. (2014). Mesoporous TiO_2_ implants for loading high dosage of antibacterial agent. *Appl. Surf. Sci.* 303, 140-146. [doi:10.1016/j.apsusc.2014.02.111](https://doi.org/10.1016/j.apsusc.2014.02.111)

Sun Y., Sun X., Li X., Li W., Li C., and Zhou Y. (2021). A versatile nanocomposite based on nanoceria for antibacterial enhancement and protection from aPDT-aggravated inflammation via modulation of macrophage polarization. *Biomaterials* 268, 120614. [doi:10.1016/j.biomaterials.2020.120614](https://doi.org/10.1016/j.biomaterials.2020.120614)
